# Ethnic differences in sedentary behaviour in 6–8-year-old children during school terms and school holidays: a mixed methods study

**DOI:** 10.1186/s12889-019-6456-3

**Published:** 2019-02-04

**Authors:** Liana C. Nagy, Maria Horne, Muhammad Faisal, M. A. Mohammed, Sally E. Barber

**Affiliations:** 1 University of Bradford, Faculty of Health Studies, Richmond Road, Bradford, BD7 1DP England; 2 Bradford Institute for Health Research, Bradford Teaching Hospitals Foundation Trust, Bradford, BD9 6RJ England; 3 Oxford Brookes University, Faculty of Health and Life Sciences, Jack Straw’s Lane, Oxford, OX3 0FL UK; 4 University of Leeds, School of Healthcare, Leeds, LS2 9JT England; 5 Yorkshire & Humberside Academic Health Sciences Network, Wakefield, UK

**Keywords:** Sedentary behaviour, sitting, Physical activity, activPAL, Ethnicity

## Abstract

**Background:**

Sedentary behaviour (SB) in childhood is a major public health concern. Little is known about ethnic differences in SB during school and holiday weeks among White British (WB) and South Asian (SA) children, which this study aims to address through investigating inclinometer measured SB and exploring reasons for child engagement in SB.

**Methods:**

A mixed methods study, comprising of a quantitative investigation with 160, 6–8 years old children and a qualitative study with a subsample of 18 children, six parents and eight teachers was undertaken. Children of WB and SA ethnicity in three schools were invited to wear inclinometers for seven school terms (summer/winter/spring) and seven holidays (winter/spring) days during July 2016–May 2017. Total SB, SB accumulated in bouts > 30 min and breaks in SB were explored using multivariate linear mixed effects models which adjusted for wear time, sex, deprivation, overweight status, season, term, weekday and school.

Nine focus groups and two interviews were carried out using the Theoretical Domains Framework to explore SB perceptions among parents, teachers and children. Data were analysed using the Framework Approach.

**Results:**

104/160 children provided 836 valid days of data. Children spent on average eight hours of SB/day during term time and holidays, equating to 60% of their awake time, and had on average 111 SB breaks /day. SA children had 25 fewer SB breaks/ day when compared to WB (*p* < 0.001). Perceived reasons for engagement in SB included: boredom, enjoyment of screen activities (by children), parenting practices, curriculum pressures (by teachers), the need to sit down and learn, and child’s preference for screen activities (by parents).

**Conclusions:**

Children spent 60% of their awake time being sedentary, regardless of ethnicity or school term. There were no significant ethnic differences for any of the SB outcomes except for breaks in SB. Interventions aimed at reducing SB should consider involving parents and teachers and should focus on increasing breaks in SB, especially for SA children, who are at a higher risk of cardio metabolic ill health.

## Background

The importance of investigating sedentary behaviour (SB) in children has been justified by the growing body of evidence linking high levels of SB to poor child development outcomes, lower academic achievement, and unfavourable cardio-metabolic risk factors in later life [1–4]. Nonetheless, inconsistencies in research findings linking SB to poor health outcomes, as well as inconsistencies in establishing the determinants of SB in children, have been noted in the literature [5, 6] and are related to the methodological quality of some studies as well as the complexity of identifying the direction and interplay between determinants of SB. Furthermore, less is known about ethnic differences in SB in child populations [7, 8] and to the best of our knowledge, no European studies have investigated SB during school holidays, which constitutes 20% of a calendar year. A recent systematic review [9] reported that children spend over half of their unstructured afterschool time in SB. Only one Japanese study has considered SB during school holidays and identified significantly higher accelerometer measured SB during school holidays compared to school term [10]. Within the existing literature, the term “level of SB”, depicts the amount of SB within a particular time window (whole day, before school, after school etc) and this has been classified as high or low without specific thresholds to define high and low SB. The SB research using inclinometry has started to focus on how total SB was accumulated, whether from short uninterrupted periods or prolonged bouts (over 30 min) and this is particularly important since longer bouts of SB have been found to be more damaging to cardiovascular health [11, 12]. Furthermore, minority ethnic groups have been found to have higher levels of SB and lover levels of PA [13]; ethnic differences are also seen in the prevalence and risk of chronic conditions [14, 15]. Specifically, evidence supports that, compared to White Europeans, SA have an increased risk of cardiovascular health and type-2 diabetes; for example the prevalence of diabetes is six time higher among SA groups [16, 17].

The aim of the study was to investigate the extent to which SB, measured via inclinometry, differed between White British (WB) and SA, 6–8-year-old children during school terms and school holidays and to explore perceived reasons for child engagement in SB.

Very few studies have explored reasons for engagement in SB and to our knowledge none have adopted a mixed method approach to ethnic differences in SB in primary school age children.

## Methods

### Ethical approval

Institutional approval from the University of Bradford Ethical Committee (E 536 06/06/2016) was granted for the study.

### Design

We used a mixed methods approach [18] comprising of a cross-sectional study with quantitative data collection at five time points and a qualitative study consisting of interviews and focus groups with parents, children and teachers. First, quantitative data was used to determine ethnic differences in activPal (PAL Technologies Ltd., Glasgow, UK) measured SB. At the same time, qualitative, interviews and focus groups were undertaken to determine perceived reasons for engagement in SB.

### Recruitment and consent

A total of 492 children, of WB and SA ethnicity, aged 6–8-year-old from three schools in West Yorkshire, UK were invited to participate in the study investigating SB, which commenced in July 2016 and ended in May 2017. Different groups of children were invited at three different time points: summer, winter and spring. Signed parental consent was gained first followed by child assent before any data collection.

### Procedures and measurements

Quantitative data collection commenced with the summer term 2016, followed by winter term 2017, winter holiday 2017, spring holiday 2017 and spring term 2017 for one week of activPal wear for each data collection period. The device was placed on the anterior side of the right thigh, in a nitrile sleeve, wrapped and attached using hypo allergic medical dressing making the device waterproof, secured on the leg and allowing for 24 h protocol.

Measurements included height (wall mounted standiometer: Seca UK, Birmingham UK), weight and body composition (measured with Tanita scales TBF-300 MA, Tokio, Japan and Tanita scales BC-418 MA, Tokio, Japan). Body mass index (BMI) z-scores were calculated for each participant using the British growth reference [19]. Weight categories (normal weight, overweight, obese) were derived from BMI percentiles using Freeman et al. recommendations [20].

Parents completed a demographics questionnaire, which included postcode details, child date of birth and child ethnicity. Parents were asked to complete sleep diaries with their children on a daily basis. The index of multiple deprivation decile (IMD) was generated from individual postcodes and collapsed in three levels of socioeconomic status (SES): low for IMD 1–2, medium for IMD 3–5 and high for IMD greater than 5. The outcomes of interest were total SB, minutes, SB minutes in bouts ≥30 min, number of bouts ≥30 min, number of breaks in SB and perceived reasons for high levels of SB.

### Qualitative data procedures

All parents whose children participated in the summer data collection and all teachers working with 6–8-year-old children in each school were invited to take part in the qualitative part. Consent was given at the time of focus group. Three boys and three girls with valid data were selected from each school and invited to participate in a focus group. The focus group and interview guides were developed using the Theoretical Domains Framework (TDF) to elicit components which need to be addressed in order to change behaviour [21].

The focus group schedules were refined through four separate pilot tests carried out with a WB 7-year-old boy, his parent, a SA parent and a WB primary school teacher working in a predominantly SA school. The pilot tests were not included in the analysis. Minor changes were made to the questions such as changing some wording for the children’s focus group. For example, instead of sedentary behaviour, the term “sitting” was used. A list of questions used in the focus group can be found in Appendix 1.The focus groups and interviews were conducted in a manner consistent with recommendations made by Kruger and Casey [22].

All focus groups and interviews were carried out in English by LN, audio-recorded and transcribed verbatim.

### Quantitative data analysis

ActivPal data were downloaded using the manufacturer’s software (activPal3™ Professional v.7.2.32, PAL Technologies Ltd., Glasgow, UK) which generated three Microsoft Excel files (EventMarker, Events.csv and EventsXYZ.csv), one “datx” file, one “def” file and one “pal” file for each device. The “csv” EventsXYZ files which contain the raw data were processed in Stata 13 [23] and used an algorithm designed for a 24-h protocol [24] to remove non-wear. As a quality control measure, several processed files were statistically and visually examined individually for plausibility of sleep/non-wear classification [25]. As only 30% of children returned completed sleep diaries, none were used in order to reduce subjectivity. A day was considered valid if it had minimum of 10 and a maximum 18 h of valid wear time. For a child to have had their data included in the analyses they had to have had at least three valid week days and at least one valid weekend day during school term and/or school holidays.

Data were analysed using multivariate linear mixed effects mixed modelling, assuming no interaction effects. A total of 9 models were built: one model for each of the three outcome variables in three data sets (whole data set, term data set and holiday data set). The covariates used were: wear time, sex, SES, weight status, weekend, occasion (holiday/term), season and school; the covariate of interest was ethnicity. Age was not included in the model since the age variation was minimal. Child was entered as a random effect. Statistical significance was set at < 0.05.

### Qualitative data analysis

The transcripts were anonymised and pseudonyms used. Data were analysed using Framework approach [26] with the TDF, as the indexing scheme to explore SB perceptions from a child, parent and teacher point of view. LN coded all transcripts using NVIVO11 [27] to code and manage the data. Codes were then discussed with the research team and together developed the indexing scheme, which was used by the first author to chart data. Discrepancies were discussed between two authors and consensus was reached through discussion. Data was then lifted from the text and placed under the thematic analytical working framework (the charting stage) headings after which key characteristics were analysed subtracting meaning, developing associations and explanations within context.

## Results

### Quantitative results

Out of 160 children who had parental consent and participated in the study, 104 (Table 1) had valid data which generated 836 valid days of term and holiday days.

**Table 1:** Demographics of the children with valid data

	All children (*n* = 104)	WB children (*n* = 60)	SA children (*n* = 44)
Age (years), mean(SD)	7.51 (0.52)	7.48(0.50)	7.50 (0.54)
Male (%)	51 (49.04)	29 (48.33)	22 (50.00)
Low SES (%) Medium SES (%) High SES (%)	44 (42.31) 45 (43.27) 15(14.42)	9 (15.00) 36 (60.00) 15 (25.00)	35 (79.55) 9(20.45) 0 (0.00)
Normal weight (%) Overweight (%) Obese (%)	78 (75) 11 (10.58) 15 (14.42)	46 (76.67) 9 (15) 5 (8.33)	32 (72.73) 2 (4.55) 10 (22.73)
zbmiuk (SD)	0.32 (1.20)	0.30 (1.03)	0.34 (1.40)

zbmiuk – child bmi z scores based on the British growth reference (UK90)

On average children were 7.51 years of age and the male/female ratio was close to one (51/53 = 0.96). The ethnic composition of the sample was representative of the area [28], 58% WB and 42% SA. There was a high percentage (42%) of children from the lowest SES status (IMD1–2) and the majority of them were SA. The majority of children were of normal weight (75%) and the percentage of overweight/obese was 25% [29]. A higher percentage of SA children were obese (22.73%) compared to WB (8.33). Across the whole data set (terms and holidays) children had an average of 814 (SD = 41) minutes of valid wear. Sixty percent (490 min) of this was spent in SB and124 (SD = 101) minutes (24%) of the total SB was generated from long bouts ≥30 min. On average children broke their SB 111 (SD = 19) times per day which generated 111 bouts of SB and 2 were bouts ≥30 min (Table 2).

**Table 2:** Unadjusted averages for wear time and SB outcomes in three data sets

	Valid days	Wear time minutes (mean, SD)	Total SB minutes (mean, SD)	% of total SB out of wear time	SB in bouts ≥ 30 min (mean, SD)	% of short and long bouts out of total SB	Number of breaks in SB (mean, SD)
Whole dataset *N* = 104	836	814 (41)	490 (56)	60	124 (101)	74 26	111 (19)
Term time *N* = 85	519	820 (44)	492 (55)	60	116 (58)	76 24	112 (17)
Holiday *N* = 48	317	804 (42)	488 (71)	61	136 (59)	71 29	110 (22)

After adjusting for wear time (divided in tertiles), sex, deprivation, weight status, season, occasion (term/holiday), weekday and school, no statistically significant (*p* < 0.005) differences were found between WB and SA children except in for one aspect of SB: SA children have 25 fewer breaks in SB compared to WB children(*p* < 0.01).

Other statistically significant differences relate to seasonality, SES and school as presented in Table 3 (Results from statistical models based on the whole data set).

**Table 3:**
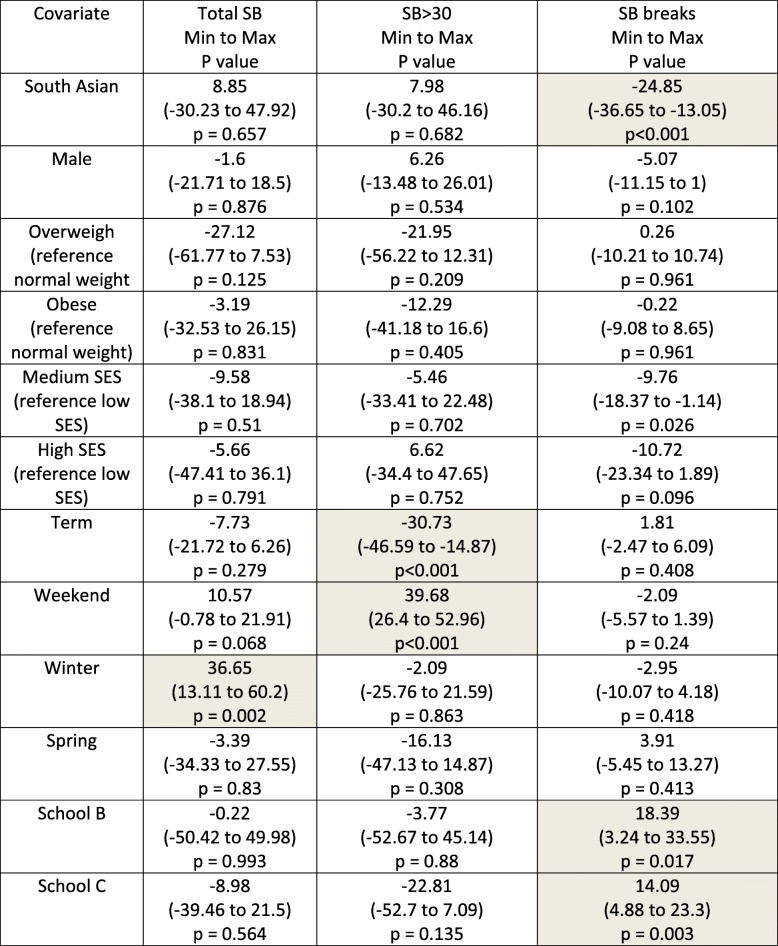
Model coefficients for three SB outcomes based on the whole data set

The models for term time showed the same trend in ethnicity as for the whole data set model: no significant ethnic differences for any aspect of SB except for break in SB. SA children have 27 fewer SB breaks compared to WB children (Table 4).

**Table 4:**
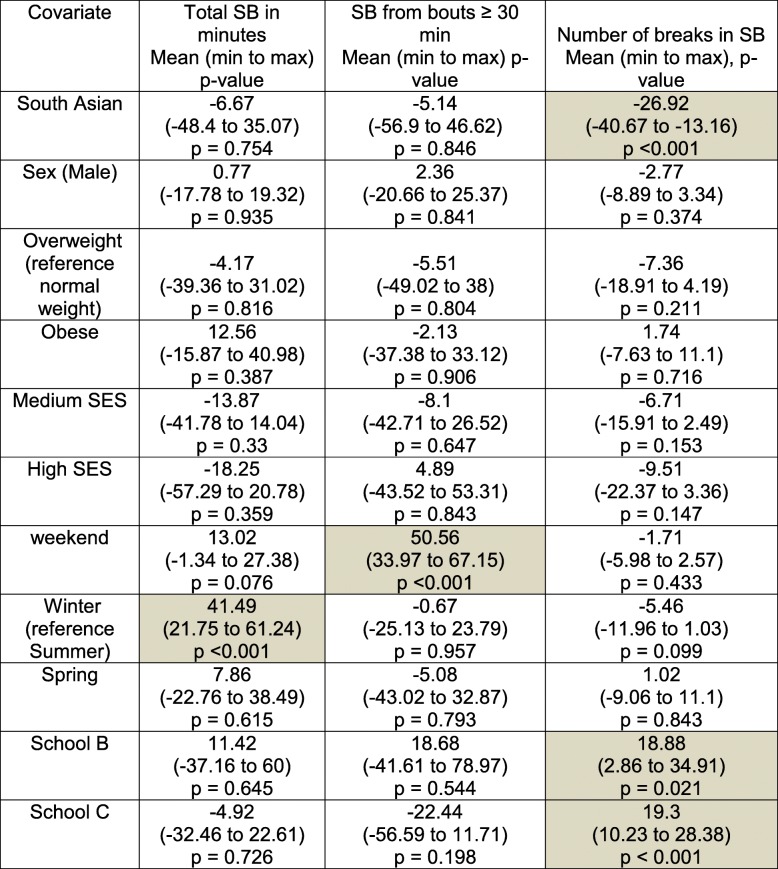
Model coefficients for three SB outcomes based on school terms data

The trend for fewer SB breaks for SA children is observed in the holiday’s model (Table 5) with 19 fewer breaks (*p* < 0.05). Except breaks in SB, there are no other ethnicity differences for any SB outcomes.

**Table 5:**
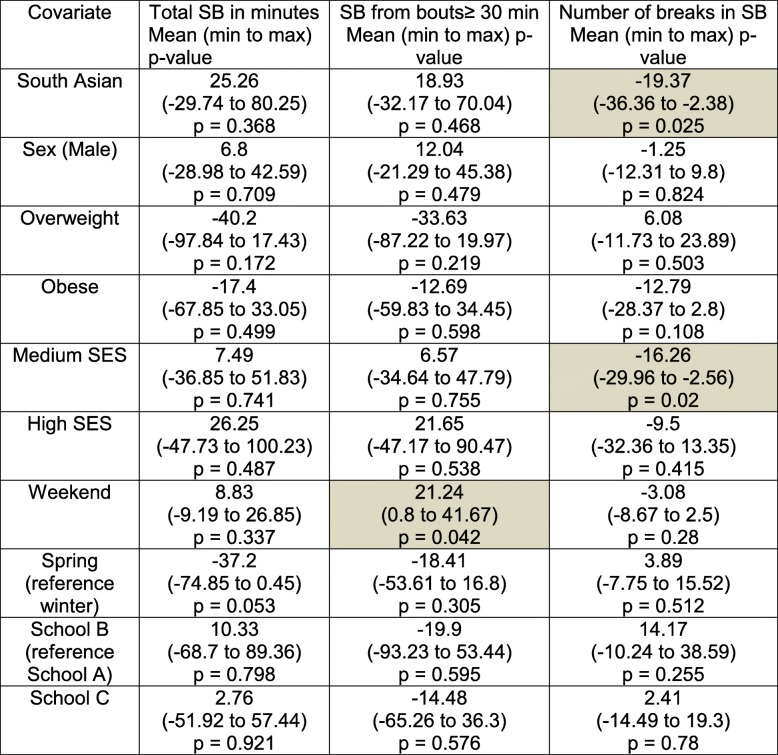
Model coefficients for three SB outcomes based on holidays data

### Qualitative results

Eighteen children, six parents and eight teachers participated in eight focus groups and 2 interviews. All meetings were intended to be focus groups but due to participant availability, two of them became interviews. Focus groups characteristics are provided in Table 6.

**Table 6:** Focus groups and interviews participants’ characteristics

Focus groups(FG) and interviews (*n* = 10)	School (*n* = 3)	School Index of Multiple Deprivation decile	Participants (*n* = 32)	FG and interview Index of Multiple Deprivation decile	Participants’ ethnicity
FG1	A	4	Children n = 6	4,4,6,6,8,8	WB
Interview 1	A	4	Parents n = 1	8	WB
FG2	A	4	Teachers n = 3	n/a	WB
FG3	B	1	Children n = 6	1,1,1,1,3,3	SA
FG4	B	1	Parents n = 2	3,1	SA
FG5	B	1	Teachers *n* = 2	n/a	WB
Interview 2	B	1	Teachers n = 1	n/a	WB
FG6	C	2	Children n = 6	2,2,4,4,5,5	WB and SA
FG7	C	2	Parents n = 3	2,5,5	WB
FG8	C	2	Teachers n = 2	n/a	WB

Although the whole qualitative exploration was in depth and explored numerous aspects of SB, this article will report specifically on the reasons for child engagement in SB.

Child, parent and teachers’ *perceived* reasons were mapped onto several TDF components: emotion, social influences, and professional role. Teachers and children perceived enjoyment and child’s preference for screen viewing activities as a reason for child engagement in SB, whilst some parents described screen viewing activities as an addiction:

*“I know [child’s name], if I wouldn’t say he can’t have it …he would be on it 24 hrs, take it to bed and sleep with it.” (WB parent, School C).*

All parents felt that learning requires sitting and one cannot be focused if “on the move”.

*“They are somewhat more focused if they are sat in one place so they can focus on what they are doing instead of just walking around.”* (WB Parent, School A).

Intra- personal reasons for SB, such as laziness, boredom and enjoyment to sit for screen viewing activities were aspects identified by boys and girls as reasons for engagement in SB:

*“They are not active, they don’t like jumping and running, they just like sitting on the sofa and watching TV, they might have a habit of sitting.”* (SA Girl, School B).

*“And if you lazy you keep on playing with your phone”* (SA Boy, School B).

Children reported numerous circumstances when they were told to sit which included classroom time, visiting grandparents, going to church or mosque, eating and or watching TV. Spending time at the mosques on a regular basis for SA children appeared to be the only ethnic difference for reason to engaging in SB.

Teachers felt that the child’s level of SB was a result of the family’s lifestyle, for example if the child had active parents, the child would be less sedentary. Teachers in predominantly SA schools perceived SA parents as overprotecting of their children and not seeing the importance of PA.

*“There are lots of children who would stay inside and there are an awful lot of parents who would say, aw so and so has got a cold today he needs to stay inside, you know he is feeling tired. I don’t think the parents see the importance of being outside in fresh air, burning energy and things like that” (School B, Teacher).*

Teachers agreed that the demands and focus on academic outcomes have become heavier in the recent years, which they felt contributed to sitting down for longer periods to complete academic work:

*“Demand. Curriculum got more difficult, the government are tracking more, they have more tests, tests are harder so what you have to do now in the classroom is much more difficult and there is other things …I think that’s why.*” (Teacher in School C).

It appears that adults have a more significant role than children in contributing to children’s engagement or disengagement with SB since children are told by adults when to sit and are guided towards activities that required sitting or not. They are therefore likely to also provide part of the solution and the means to reducing child SB.

## Discussion

The study found that WB and SA children spent 60% of their awake time in SB during term or holiday. Out of the total SB, 25% in bouts ≥30 min. There were no significant differences between WB and SA children in SB except for breaks in SB. SA children had 25 fewer breaks compared to WB children. Among the perceived reasons for engagement in SB were: enjoyment of screen viewing, boredom, laziness, parenting, curriculum demands and the need to sit for academic learning.

This study highlighted the need for the overall reduction of SB regardless of ethnicity. Sitting has been compared to smoking in the recent literature [30, 31] because of its effects on health, and if this is the case, more needs to be done to reduce it. The strength this study lies in its mixed methods design, inclusion of school holiday days in analyses and the focus on ethnic differences. The majority of studies evaluating SB are quantitative [9, 32–34] and very few are qualitative [35–37]. Fewer even have a mixed method design that allows for contextual data to explain some of the quantitative findings [38]. No European studies have yet considered SB during school holidays in any age group and within the field of physical activity and SB, studies that look at ethnic differences in primary school age children are only emerging [39].

The present study addressed the question of ethnicity in relation to SB, giving a macro view of the whole day. Specific evaluations of SB within time periods in the day that are relevant to primary school aged children might offer more insight and allow the identification of critical time periods when SB interventions are mostly needed.

A limitation of this study is the use an algorithm that was validated in adults to remove non-wear. To date there isn’t a validated method for activPal data in child populations and most studies do no report how the non-wear time was addressed or use the accelerometry rule of 0 counts exclusion [40–42]. Non-wear time needs to be addressed when processing activPal data and further research should consider developing validated methods in child populations. To increase accuracy of the results several files were visually inspected for quality control and manually processed, however further research needs to clarify how short periods of non-wear should be dealt with [25].

The sample of children who had valid data is modest (104/160) and this was due to device availability and participant compliance with wearing the device. Non- compliance with device wear was also due to low tolerance for wearing the device for some children. Some children (*n* = 33) had a rash after several days of wearing the device, which was reported to the manufacturer. No other studies have reported on this issue but further research should consider developing alternative ways to device attachment.

The cross-sectional design of the study comes with its limitations and no conclusions could be made about the determinants of SB in children of SA or WB ethnicity. To date no longitudinal study has reported results using the activPal and this is likely to do with the age of the device as the first ever study using activPal was published in 2007 [43].

All children in this study spent eight hours of their awake time in SB regardless of ethnicity which is comparable with office workers SB [44]. The qualitative data gave some insight into underlying reasons for engagement in SB but further exploration is required. Nonetheless, unpublished questionnaire data from the Born in Bradford cohort study (completed 2016/2017) with 2356 children of SA ethnicity who completed the religion section might provide possible clues. Of all the SA children who completed the religion questions, 96% identified themselves as being Muslim, 91% said that they attended Mosque/Madrassa and 85% said they attended Mosque/Madrassa on some or most days of the week (71%, most days of the week). Children spend 1–2 h daily at madrasa and it is expected they are mostly seated as this is a religion and language learning environment. The way in which prayers are done requires changes in posture but the proportion of SB accumulated during the week is unclear as no device recorded data on child SB in mosques is available.

This study did not find any significant SB differences between term and school holiday, which is contrary to previous studies [45, 46] evidencing increased SB during unstructured periods of time (after school hours or weekend). The reasons for this are unclear and it warrants further research. It was also anticipated that there would be significant ethnic differences in the total SB based on results from studies evaluating ethnic differences in SB in child populations in Bradford [8, 47, 48]. However, this was not the case for our study. SB increases with age and a possible explanation for the lack of ethnic differences is the age group of the children evaluated. Inclinometer recorded data from older children (9–10 years old) in Bradford highlighted significant ethnic differences in the total SB [47]. Twenty-five percent of children in our study were overweight and obese which is similar to data published in the UK [49]. The recent predictions by World Health Organization (WHO) on rising child overweight and obesity to 40% by 2030, if no action is taken, are relevant for both developed and developing countries. Part of the WHO strategy for reducing overweight and obesity is to reduce SB [50].

A recent systematic review reported a rise in SB as children transition to secondary school [32]. Given the findings of our study, this is concerning if the already high levels of SB are expected to rise. The results on total SB in our study are comparable with the results of the systematic review for children already at transition [32], but the studies included in the review were published between 1999 and 2015 and it is likely that due to increasing use of screen devices, the total SB may also be increasing.

A standing issue relates to guidelines and practices that contribute to increasing levels of SB. Whilst there are recommended guidelines for levels of MVPA for children there are no such guidelines for SB. In the 2016 Report Card on Physical Activity for Children and Youth [51], the results on SB has received an incomplete grade due to lack of guidelines. Our findings highlight an urgent need to address SB in school children who are as sedentary as office workers. As schools have often been identified to be optimal environments for physical activity interventions, school policies could consider adopting a benchmark for SB during school time. As adults are likely to be more significant contributors to child engagement in sedentary behaviour, interventions aimed at reducing SB should consider including parents and teachers [52].

## Conclusions

Our study has highlighted high levels of SB regardless of ethnicity and showed no significant ethnic differences in SB except for breaks in SB. Since breaks in SB are desirable for cardiovascular health, interventions aimed at reducing SB should also consider increasing breaks in SB, especially for SA children who are at a higher risk of cardio metabolic ill health in adulthood.

## Abbreviations

FG: Focus group
IMD: Index of Multiple Deprivation
MVPA: Moderate to vigorous physical activity
PA: Physical activity
SA: South Asian
SB: Sedentary behaviour
SES: Socioeconomic status
TDF: Theoretical Domains Framework
WB: White British
WHO: World Health Organisation
